# Lumboperitoneal Shunts for the Treatment of Idiopathic Normal Pressure Hydrocephalus: A Comparison of Small-Lumen Abdominal Catheters to Gravitational Add-On Valves in a Single Center

**DOI:** 10.1093/ons/opy044

**Published:** 2018-04-23

**Authors:** Madoka Nakajima, Masakazu Miyajima, Chihiro Akiba, Ikuko Ogino, Kaito Kawamura, Hidenori Sugano, Takeshi Hara, Yuichi Tange, Keiko Fusegi, Kostadin Karagiozov, Hajime Arai

**Affiliations:** Department of Neurosurgery, Juntendo University School of Medicine, Tokyo, Japan

**Keywords:** Cerebrospinal fluid, Cognitive function, Hydrocephalus, Ventriculomegaly, Gravity

## Abstract

**BACKGROUND:**

Treating idiopathic normal pressure hydrocephalus (iNPH) with lumboperitoneal shunts (LPSs) may cause cerebrospinal fluid (CSF) overdrainage.

**OBJECTIVE:**

To investigate whether LPSs, including gravitational “add-on” and programmable pressure valves (PPVs/+GVs), reduce complications and improve outcomes.

**METHODS:**

We compared PPVs/+small lumen abdominal catheters (SLs) to PPVs/+GVs using different opening pressures for supine and standing positions. We analyzed 115 patients with iNPH in 2 consequent cohorts: 48 patients receiving LPSs with PPVs/+SLs and 67 patients receiving LPSs with PPVs/+GVs. The modified Rankin Scale (mRS), Japan iNPH grading scale, Mini Mental State Examination, Frontal Assessment Battery, and CSF biomarkers were evaluated.

**RESULTS:**

Comparisons of postoperative clinical factors in 64 patients in the PPV/+SL and PPV/+GV groups using 1:1 propensity score matching revealed differences in the mean (±standard deviation) postoperative mRS (2.65 ± 1.07 vs 2.16 ± 1.02, *P* = .049) and gait disturbance scores (1.97 ± 1.03 vs 1.39 ± 0.92, *P* = .011). Thus, outcomes improved in the LPS group with the GV. Serious and nonserious adverse event rates for the PPV/+SL and PPV/+GV groups were 22.9% and 19.4% (*P* = .647) and 38% and 17.9% (*P* = .018), respectively, indicating higher rates of subdural collections for the PPV/+SL group.

**CONCLUSION:**

This is the first study to examine LPS treatment for iNPH using a GV in tandem with a PPV. Our results suggest that the CSF shunt flow volume is restricted in the standing position and maintained in the supine position, thus improving iNPH symptoms. This may reduce intracranial CSF hypotension-related complications.

**ABBREVIATIONS**
CSF: cerebrospinal fluid
FAB: Frontal Assessment Battery
GV: gravitational add-on valve
ICP: intracranial pressure
iNPH: idiopathic normal pressure hydrocephalus
iNPHGS: idiopathic normal pressure hydrocephalus grading scale
LPS: lumboperitoneal shunt
MMSE: Mini Mental State Examination
mRS: modified Rankin Scale
OD: overdrainage
PPV: programmable pressure valve
SD: standard deviation
SL: small-lumen
VPS: ventriculoperitoneal shunt

Idiopathic normal pressure hydrocephalus (iNPH), a disease with uncertain etiology that characteristically afflicts older adults, is characterized by cognitive decline, gait and balance impairments, and urinary incontinence.^1,2^ iNPH is commonly treated with cerebrospinal fluid (CSF) shunts, as this is the only treatment with clear evidence of effectiveness.^3^ Although the mechanism for recovery following shunt treatment remains unclear, research suggests that shunts improve CSF clearance and adjust the intracranial pressure (ICP).^4^ Subsequent studies have demonstrated that lymphatic transport is controlled by the brain's arousal level.^5,6^ The volume of the interstitial space in the brain expands significantly during sleep or anesthesia when compared with the awake state.^7,8^ CSF shunts may also facilitate the excretion of brain extracellular metabolites, which include neurotoxic proteins such as amyloid beta (Aβ) and phosphorylated tau (*p*-tau).^9,10^ A previous study reported elevated Aβ38 and Aβ42 levels following shunt implantation, likely owing to improved CSF clearance after surgery.^10^

Ventriculoperitoneal shunt (VPS) implantation is currently the standard treatment for patients with iNPH.^11^ In Japan, however, a lumboperitoneal shunt (LPS), which is a less invasive treatment, is used more frequently.^12^ LPS treatment has been recognized as an effective approach for iNPH,^13^ though its higher revision rates compared with VPS and more-frequent complications due to CSF overdrainage (OD) are problematic.^14^ Although the mechanisms are unclear, OD seems to occur more frequently in tall and thin individuals. Some authors contend that the siphon effect in the standing position has the strongest influence on this mechanism.^15-17^ The programmable pressure valve (PPV), in which the pressure is adjusted according to the individual's ICP and body constitution, is known to be more effective than fixed pressure valves for preventing OD complications.^18,19^ However, the PPV cannot control the CSF shunt flow volume when there is a change in posture or an increase in ICP due to coughing or straining.^20,21^ This is a limitation that requires a solution.

Various anti-siphon devices that control CSF shunt flow volume are available. They include siphon guards (Codman and Shurteleff; Johnson and Johnson, New Brunswick, New Jersey), small-lumen abdominal catheters (SL 43555; Medtronic Neurosurgery, Medtronic Inc, Dublin, Ireland), and gravitational “add-on” valve systems (GV; Aesculap-Miethke, Tuttlingen, Germany).^22^ The inner diameter (0.7 mm) of the SL catheter is smaller than that of the conventionally used abdominal catheter. The increased resistance of the SL catheter aims to prevent OD.^12^ However, the siphon guard effect of the SL catheter is not necessarily effective as a short abdominal catheter for LPS, as it depends on the catheter length. Some studies have reported the effectiveness of GVs for the prevention of OD in devices that automatically increase shunt pressure with changes in posture.^23^ The gravitational unit gradually adds resistance to the pre-set opening pressure when the valve is raised from an angle of 0° toward the upright position at an angle of 90°.

While facilitating the excretion of brain extracellular metabolites, it is important to maintain a CSF flow of sufficient volume to increase the clearance of these proteins while avoiding OD complications. We hypothesized that CSF shunt with GV for iNPH would clear brain extracellular metabolites more efficiently during sleep and in the supine position. Shunt treatment guarantees a larger CSF shunt flow volume in the supine position, which indicates that shunt systems with GV can be developed to remove brain extracellular metabolites. However, to our knowledge, no studies have yet investigated the efficacy LPS with a GV. Therefore, we evaluated whether LPS treatment using a shunt system with a GV installed in tandem with a PPV would reduce complications arising from OD and improve patient outcomes.

## METHODS

### Study Design

**FIGURE 1.**
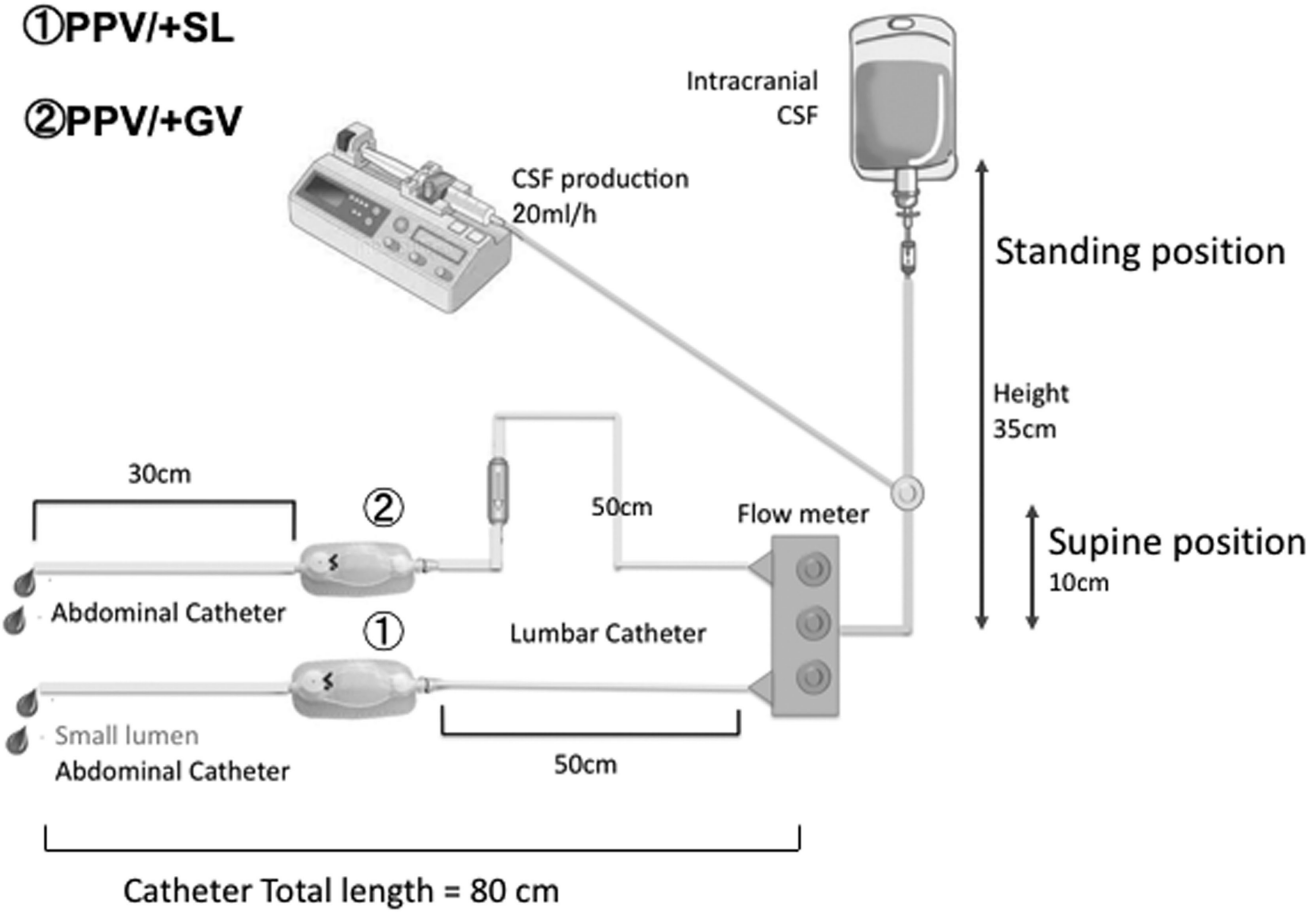
Shunt system experiments. Based on previous studies, we set the standing (vertical) pressure at 35 cmH_2_O and the supine (horizontal) position pressure at 10 cmH_2_O. We simulated CSF “production” at 20 mL/h by injecting H_2_O using a syringe pump connected to the main hydrostatic pressure column. We measured the flow from the 2 types of systems under the 2 different postural conditions (vertical and horizontal) by measuring total discharge for 10 min at 5 performance levels, as follows: 0.5 (15-35 mmH_2_O), 1.0 (35-55 mmH_2_O), 1.5 (90-110 mmH_2_O), 2.0 (145-165 mmH_2_O), and 2.5 (200-220 mmH_2_O).

**FIGURE 2.**
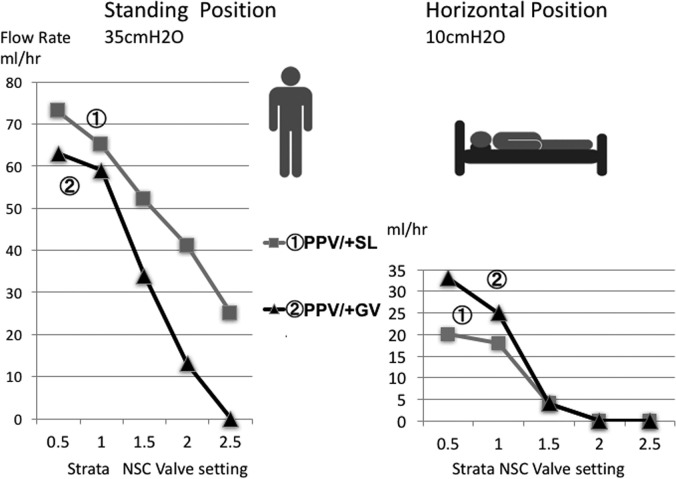
Comparison between the small-lumen abdominal catheter and gravitational add-on valve. We measured the flow rate (mL/h) at simulated standing (35 cmH_2_O) and supine (10 cmH_2_O) positions for the 5 performance levels of the Strata NSC valve, ranging from 0.5 to 2.5. When the PPV and GV were placed in tandem and the pressure was set at level 2.5 (22.5 cmH_2_O) plus 15 cmH_2_O, the flow volume was 0 mL in the standing position. In this position, the GV led to a greater reduction in flow volume than did the small-lumen abdominal catheter at all settings, while greater flow volume was obtained at the lower level settings of 0.5 and 1.0 in the supine position.

We implanted a GV, which is used to maintain higher CSF flow in the supine position, and a PPV set to low pressure in a sequential column to create an LPS. We compared this device to an SL catheter. We conducted a preliminary experiment to test a system using the siphon effect created by the 2 different types of devices to prevent excessive CSF outflow. A previous study reported that patients with iNPH require a mean (±standard deviation [SD]) vertical effective opening pressure of the entire shunt system of 27.5 ± 3.3 cmH_2_O, while patients with congenital hydrocephalus require a pressure of 36.3 ± 23.3 cmH_2_O, and patients with malresorptive hydrocephalus require a pressure of 35.9 ± 20.4 cmH_2_O.^22^ We therefore set the standing (vertical) pressure at 35 cmH_2_O (close to the pressures established above) and the supine (horizontal) position pressure at 10 cmH_2_O. The results of the experiment indicated that GVs placed into a column in sequence limit CSF flow to a greater extent while the patient is in the standing position, and maintain flow while the patient is in the supine position (Figures 1 and 2).

We compared LPS treatment in individuals with iNPH using 2 different shunt systems designed to prevent OD. Two consecutive groups of patients were treated. The first group was treated with an SL catheter attached to a PPV, which represented the best available standard treatment (PPV/+SL group).^12^ The second group was treated with a GV in tandem with a PPV with different opening pressures (PPV/+GV group). We compared the PPV/+GV group to the PPV/+SL group, which was used as a control group. The components listed in sequence from proximal to distal were as follows: PPV/+SL group, lumbar catheter + Strata NSC valve + SL catheter (43555); and PPV/+GV group, lumbar catheter + GV “shunt assistant” 0-15 cmH_2_O (Aesculap-Miethke) + Strata NSC valve + abdominal catheter (27536).

### Patients

**FIGURE 3.**
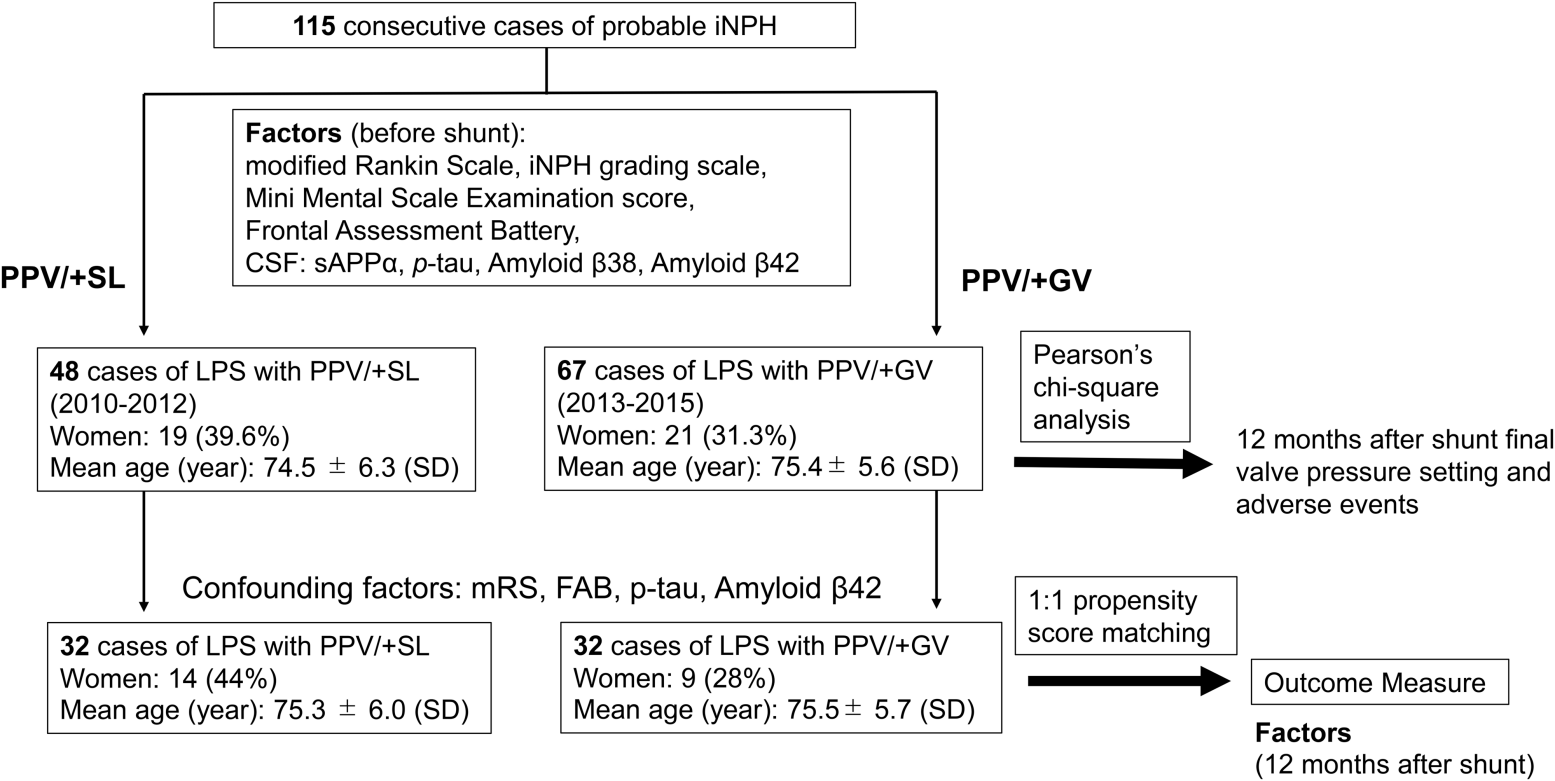
Subject flow diagram. GV = gravity add-on valve, iNPH = idiopathic normal pressure hydrocephalus, LPS = lumboperitoneal shunt, MMSE = Mini Mental State Examination, PPV = programmable pressure valve, *p*-tau = phosphorylated tau, sAPPα = soluble amyloid precursor protein alpha, SL = small-lumen abdominal catheter.

The patients were reviewed retrospectively. The patients in this cohort received an LPS after attending consultations in our department and were suspected of having iNPH based on neurological manifestations and magnetic resonance imaging. Our evaluation criteria were consistent with the Japanese guidelines for iNPH.^24,25^ We compared the following 2 iNPH patient groups (n = 115 in total): 48 patients treated with LPS with PPV/+SL (19 women; mean age ± SD, 74.5 ± 6.3 yr; from 2010 to 2012), and 67 patients treated with LPS with PPV/+GV (21 women; 75.4 ± 5.6 yr; from 2013 to 2015; Figure 3 and Table 1). Twelve months after shunt implantation, we evaluated the patients using the modified Rankin Scale (mRS),^26^ iNPH grading scale (iNPHGS),^27^ Mini Mental State Examination (MMSE),^27^ and Frontal Assessment Battery (FAB).^29^ We also measured CSF biomarkers (soluble amyloid precursor protein [sAPPα], Aβ38, Aβ42, and *p*-tau).^30^ All patients provided prior written consent for their participation in the study, which was conducted after ethics committee approval.

**TABLE 1.** Characteristics at Baseline Data for iNPH Patients Before Lumboperitoneal Shunt

	Unmatched (bivariate)				Matched 1:1			
	PPV/+SL	PPV/+GV	Total	*P*-value	PPV/+SL	PPV/+GV	Total	*P*-value
Patients [number]	48	67	115		32	32	64	
Sex: women [number (%)]	19 (40%)	21 (31%)	40 (35%)	.360	14 (44%)	9 (28%)	23 (36%)	.193
Age (yr) [mean ± SD]	74.5 ± 6.3	75.4 ± 5.6	75.0 ± 5.9	.921	75.3 ± 6.0	75.5 ± 5.7	75.4 ± 5.8	.619
BMI (kg/m^2^) [mean ± SD]	23.5 ± 3.6	24.1 ± 3.4	23.8 ± 3.5	.498	23.3 ± 3.6	23.9 ± 3.1	23.6 ± 3.3	.627
DESH [number (%)]	38 (79%)	56 (84%)	94 (82%)	.546	26 (81%)	26 (81%)	52 (81%)	1.000
Clinical findings [mean ± SD]								
mRS	3.04 ± 0.80	2.76 ± 0.68	2.88 ± 0.74	*.042	3.09 ± 0.86	2.81 ± 0.62	2.95 ± 0.74	.088
iNPHGS-total	5.96 ± 2.42	5.72 ± 2.22	5.82 ± 2.30	.538	6.19 ± 2.64	5.47 ± 2.05	5.76 ± 2.38	.244
Gait disturbance	2.42 ± 0.82	2.24 ± 0.79	2.32 ± 0.80	.313	2.47 ± 0.92	2.19 ± 0.75	2.33 ± 0.84	.249
Cognitive impairment	1.92 ± 1.05	1.73 ± 0.90	1.81 ± 0.97	.227	2.03 ± 1.09	1.71 ± 0.69	1.87 ± 0.92	.133
Urinary incontinence	1.73 ± 1.14	1.83 ± 1.03	1.79 ± 1.08	.729	1.78 ± 1.21	1.74 ± 0.97	1.75 ± 1.09	.853
Neuropsychological Test [mean ± SD]								
MMSE	22.6 ± 5.0	21.7 ± 6.5	22.1 ± 5.9	.635	21.8 ± 5.6	22.6 ± 4.7	22.2 ± 5.2	.751
FAB	12.0 ± 3.4	10.3 ± 3.9	11.0 ± 3.8	*.017	11.4 ± 3.9	10.9 ± 2.8	11.1 ± 3.4	.339
CSF biomarker [mean ± SD]								
*s*APPα (ng/mL)	146.7 ± 75.4	155.3 ± 73.4	151.7 ± 74.1	.471	151.9 ± 84.3	148.9 ± 70.4	150.4 ± 77.1	.973
*p*-tau (pg/mL)	23.8 ± 10.2	31.1 ± 14.5	28.1 ± 14.5	*.005	24.4 ± 10.2	25.7 ± 7.8	25.1 ± 9.1	.282
Aβ38 (pg/mL)	2261 ± 1711	2500 ± 1297	2414 ± 1457	.102	2453 ± 1748	2483 ± 1503	2468 ± 1617	.582
Aβ42 (pg/mL)	383 ± 209	561 ± 234	487 ± 240	*<.001	416 ± 202	484 ± 157	459 ± 172	.182
Aβ38/Aβ42	6.45 ± 4.55	5.05 ± 3.48	5.55 ± 3.94	.088	6.49 ± 3.58	5.64 ± 4.30	5.86 ± 3.92	.338
Aβ42/*p*-tau	18.12 ± 10.28	21.25 ± 11.14	19.94 ± 10.85	.175	20.25 ± 9.99	20.75 ± 9.08	20.50 ± 9.81	.914

PPV: programmable pressure valve, SL: small inner-lumen abdominal catheter, GV: gravitational “add-on” valve, BMI: body mass index, DESH: disproportionately enlarged subarachnoid-space hydrocephalus, mRS: modified Rankin Scale, iNPHGS: idiopathihc normal pressure hydrocephalus grading scale, MMSE: mini mental scale examination, FAB: Frontal Assessment Battery, *s*APPα: soluble amyloid precursor protein alpha, *p*-tau: phosphorylated-tau, Aβ: amyloid beta.

Binomial logistic regression. Preservation prediction value was obtained by calculating the score with probability *P* and producing preoperative numerical values between the PPV/+SL and PPV/+GV groups. Propensity score: 0.186-0.863. *P < .05.

### Procedure

**FIGURE 4.**
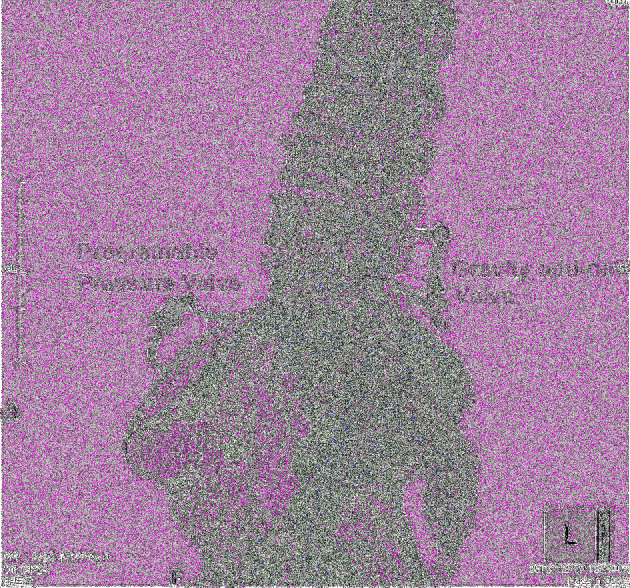
A lumbar 3-dimensional image obtained after LPS implantation with a gravity add-on valve and a Strata NSC programmable pressure valve.

Neurologists or neurosurgeons screened the patients, and 2 neurosurgeons selected the patients for surgery. Surgeons with extensive experience in the procedure conducted both types of surgical procedures. No significant technical difference was identified between the LPS with PPV/+SL and the LPS with PPV/+GV implantation procedures.^12^ Implantations were conducted under general or spinal anesthesia. In the LPS technique, approximately 15 cm of the spinal catheter was inserted through the L3/4 or L2/3 interlaminar space into the lumbar subarachnoid space using the paramedian approach. In the PPV/+GV group, after using the step-down connector, the GV was placed in the lower back so that it would be vertical when the patient was in the standing position (Figure 4). Once the L-shaped connector (Aesculap-Miethke) was applied, a subcutaneous tunnel was made to the ventral side, the abdominal catheter was passed through it ventrally, and the PPV was placed subcutaneously on the ventral side. The abdominal catheter, trimmed to about 30 cm, was inserted into the abdominal cavity after being connected to the PPV.

### Valve Pressure Adjustment Protocol

The initial pressure for the shunt system was set to its highest level of 2.5 (22.5 cmH_2_O). We checked the function of the shunt if no improvement in the patient's clinical symptoms were observed, when the high convexity and medial subarachnoid spaces were tight, or when acute callosal angles were observed.^12^ We lowered the pressure setting by 1 step (0.5 level) if symptoms did not improve at all 1 wk after the operation. If improvement was observed, but was deemed insufficient (half or less of the effects observed in the tap test, except when there was a steady improvement), we lowered the pressure setting further by 0.5-level intervals, with careful consideration of the patient's safety range. The pressure setting was increased immediately if a symptomatic subdural hematoma related to OD was found.

### CSF Analysis

We obtained lumbar CSF before and after the LPS procedure. All CSF samples were aliquoted and stored in polypropylene tubes at –80°C until biochemical analysis.^28^ Shunt reservoir and lumbar CSF biomarkers were also compared.^9,10^ CSF biomarkers included sAPPα (Human sAPPα Assay Kit; IBL No. 27719; Immuno-Biological Laboratories Co, Ltd, Takasaki, Japan), Aβ38 (Human Amyloid β1-38 Assay Kit, IBL No. 27717; Immuno-Biological Laboratories Co, Ltd), Aβ42 (Innotest β amyloid 1–42; Innogenetics, Ghent, Belgium), and *p*-tau (Innotest phospho tau-181p; Innogenetics). Immunosorbent assays were used for the rest of the biomarker measurements (Table 1).

### Outcome Measures

We compared the mRS, iNPHGS, MMSE, and FAB scores, and sAPPα, Aβ38, Aβ42, and *p*-tau concentrations, as well as the presence of any complications before and 12 mo after LPS.

### Statistical analysis

Patients who underwent the PPV/+GV implantation procedure were matched using 1:1 propensity score matching to patients who underwent the PPV/+SL procedure. Propensity scores were calculated for background factors comprising preintervention mRS and FAB scores, and *p*-tau and Aβ42 levels (Table 1). We used nonparametric methods for the analyses, including Mann–Whitney *U*-tests and Pearson's chi-squared tests, to identify changes from baseline. We used an analysis of covariance to reveal differences in the changes between the patient groups.

Adverse events were analyzed using a safety analysis set that included all patients who received an LPS. The significance level was set at a 2-sided *P* = .05. Statistical data were analyzed using SPSS version 22 (IBM Inc, Armonk, New York).

## RESULTS

### Clinical Findings

**FIGURE 5.**
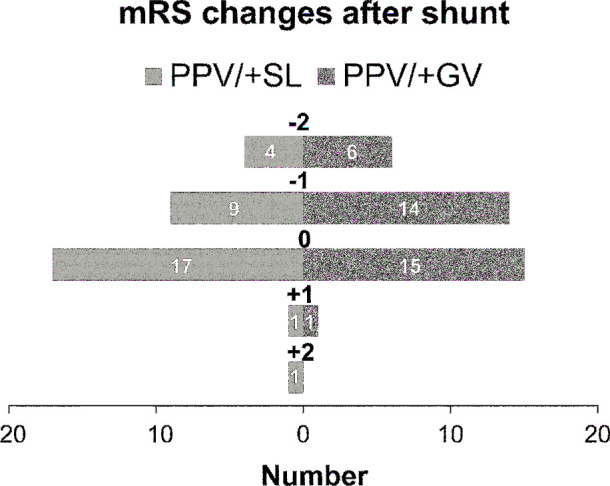
mRS score changes after LPS implantation.

The final programmable median valve pressures for the PPV/+SL and PPV/+GV groups were 1.5 (interquartile range, 1.0-2.0) and 1.0 (interquartile range, 0.5-1.5), respectively. We succeeded in using lower pressures on the pressure-adjustable GV. A comparison of postoperative clinical factors in the 64 patients in the PPV/+SL and PPV/+GV groups that was adjusted and 1:1 propensity score matched for confounding variables indicated significant differences in the mean (±SD) postoperative mRS scores (2.65 ± 1.07 vs 2.16 ± 1.0, *P* = .049) and iNPHGS gait disturbance scores (1.97 ± 1.03 vs 1.39 ± 0.92, *P* = .011). Therefore, the outcomes were improved in the LPS group with the GV. No statistically significant differences in the other factors were identified (Figure 5; Table 2). The *p*-tau concentration was the only CSF biomarker that was significantly different between the groups. The level of *p*-tau was 52.1 ± 30.2 pg/mL in the PPV/+SL group and 77.0 ± 41.0 pg/mL in the PPV/+GV group (*P* = .015).

**TABLE 2.** Comparisons Before and After Therapeutic Shunt Intervention

	After				Before/After (*P*-value)		
	PPV/+SL	PPV/+GV	Total	SL/GV (*P*-value)	SL	GV	Total
Patients [Number]	32	32	64		32	32	64
Clinical findings [mean ± SD]							
mRS	2.65 ± 1.07	2.16 ± 1.02	2.41 ± 1.07	*.049	*.014	*<.0001	*<.0001
iNPHGS-total	4.47 ± 2.83	3.44 ± 2.06	3.95 ± 2.51	.095	*.001	*<.0001	*<.0001
Gait disturbance	1.97 ± 1.03	1.39 ± 0.92	1.68 ± 1.01	*.011	*.001	*<.0001	*<.0001
Cognitive impairment	1.50 ± 1.14	1.09 ± 0.69	1.30 ± 0.95	.150	*.003	*<.0001	*<.0001
Urinary incontinence	1.25 ± 1.16	1.00 ± 0.80	1.13 ± 1.00	.544	*.003	*<.0001	*<.0001
Neuropsychological Test [mean ± SD]							
MMSE	23.8 ± 5.4	25.2 ± 4.1	24.5 ± 4.8	.357	*.002	*<.0001	*<.0001
FAB	13.0 ± 3.7	12.0 ± 3.3	12.5 ± 3.5	.182	*.018	.086	*.004
CSF biomarker [mean ± SD]							
*s*APPα (ng/mL)	153.9 ± 73.7	202 ± 114.7	178.9 ± 99.3	.132	.642	*<.0001	*.008
*p*-tau (pg/mL)	52.1 ± 30.2	77.0 ± 41.0	64.8 ± 37.9	*.015	*.009	*<.0001	*<.0001
Aβ38 (pg/mL)	3745 ± 2193	3421 ± 1528	3581 ± 1877	.815	*<.0001	*.003	*<.0001
Aβ42 (pg/mL)	578 ± 274	667 ± 335	623 ± 308	.372	*.003	*.005	*<.0001
Aβ38/Aβ42	6.49 ± 3.58	6.08 ± 4.12	6.28 ± 3.84	.338	.758	.432	.400
Aβ42/-*p*-tau	13.38 ± 8.48	11.28 ± 8.48	12.31 ± 7.82	.357	*.001	*<.0001	*<.0001

PPV: programmable pressure valve, SL: small inner-lumen abdominal catheter, GV: gravitational “add-on” valve, SD: standard deviation, BMI: body mass index, DESH: disproportionately enlarged subarachnoid-space hydrocephalus, mRS: modified Rankin scale, iNPHGS: idiopathic normal pressure hydrocephalus grading scale, MMSE: Mini Mental State Examination, FAB: Frontal Assessment Battery, *s*APPα: soluble amyloid precursor protein alpha, *p*-tau: phosphorylated tau, Aβ: amyloid beta. *P < .05.

### Adverse Events Associated with LPS

The incidence of nonserious adverse events was significantly different between the 2 groups (*P* = .018; Table 3). The rates of postoperative headache in the PPV/+SL and PPV/+GV groups were 29.2% (n = 14/48) and 14.9% (n = 10/67), respectively (*P* = .064). The rates of asymptomatic subdural effusions and subdural hematomas (conservatively treated) were 8.3% (n = 4/48) and 3% (n = 2/67) in the PPV/+SL and PPV/+GV groups, respectively (*P* = .203). The rates of chronic subdural hematomas requiring surgery were 3.9% (n = 2/48) and 0% in the PPV/+SL and PPV/+GV groups, respectively (*P* = .092). The proportion of patients requiring revision due to proximal catheter failure was 12.5% (n = 6/48) in the PPV/+SL group and 16.4% (n = 11/67) in the PPV/+GV group (*P* = .559). No statistically significant differences were found in the occurrence of serious adverse events between the 2 groups.

**TABLE 3.** Adverse Events

Parameter	PPV/+SL	PPV/+GV	*P*-value
No. of patients	48	67	
Serious adverse events			
No. of patients (%)	11 (22.9%)	13 (19.4%)	.647
Subdural hematoma requiring surgery	2 (4.1%)	0 (0%)	.092
Shunt tube—total events	6 (12.5%)	11 (16.4%)	.559
Shunt tube migration requiring revision	2 (4.1%)	1 (1.5%)	.375
Shunt tube rupture requiring revision	0 (0%)	1 (1.5%)	.395
Shunt tube obstruction requiring revision	4 (7.8%)	9 (13.4%)	.394
Meningitis	1 (2.1%)	1 (1.5%)	.811
Cerebral infarction	2 (4.1%)	1 (1.5%)	.375
Death	1 (2.1%)	0 (0%)	.235
Nonserious adverse events			
No. of patients (%)	18 (38%)	12 (17.9%)	*.018
Postural headache	14 (29.2%)	10 (14.9%)	.064
Asymptomatic subdural effusion and subdural hematoma (conservative treatment)	4 (8.3%)	2 (3.0%)	.203

Chi-squared test: comparison of adverse events between the PPV/+SL and PPV/+GV groups after shunt insertion. *P < .05.

## DISCUSSION

This is the first study to examine the use of GVs for LPS in individuals with iNPH, although research groups in Germany have already reported a multicenter open-label randomized parallel-group trial for VPS.^23^ The abovementioned study, called SVASONA, found that GVs reduce the risk of OD complications following VPS surgery. Selecting the appropriate opening pressure for the gravitational unit remains a critical treatment decision that is made according to the patient's body mass index. The rationale behind this setting is that the level of compensation for hydrostatic pressure (HP) changes ultimately depends on the height of the upper body and the intra-abdominal pressure (IAP). Kajimoto et al^31^ evaluated the ICP, IAP, HP, and perfusion pressure of the shunt system using telemetric sensors in 13 patients in both the supine and sitting positions. In the supine position, the mean (±SD) ICP, IAP, and HP were 4.6 ± 3 mm Hg, 5.7 ± 3.3 mm Hg, and 3.3 ± 1 mm Hg, respectively. As a result, the perfusion pressure was 2.2 ± 4.9 mm Hg. When patients were raised to the sitting position, the IAP increased to 14.7 ± 4.8 mm Hg, ICP decreased to –14.2 ± 4.5 mm Hg, and HP increased to 42.9 ± 3.5 mm Hg. Consequently, the perfusion pressure increased to 14 ± 6.3 mm Hg. These data are useful for determining LPS function parameters in the standing position. The LPS system with a PPV/+GV (0-15 cmH_2_O) has the potential to suppress the standing flow volume rate when the PPV is set to the appropriate pressure and there is sufficient supine flow volume.

### Changes in Neurological Symptoms and Complications after LPS Surgical Treatment

Here, LPS treatment with PPV/+GV reduced nonserious OD complications when compared with LPS with PPV/+SL. However, orthostatic headache, which was observed immediately after LPS, was present even in the PPV/+GV group. We surmise that orthostatic headache occurs due to CSF leakage around the spinal catheter into the epidural space in the early postoperative period.^32,33^ This is a problem unique to LPS.

Patients who were treated using GVs had a tendency of higher revision rates due to proximal catheter failure. As the GV must be placed in tandem with the PPV, we presume that tube kinking occurs more readily in front of or behind the GV when it is placed vertically. Technically, this catheter kinking could be avoided by directly fixing the GV or the L- or U-shaped connector to the fascia. We may be able to avoid these complications in the future if a device is developed that allows the GV to be placed horizontally.

### Changes in CSF Biomarkers After Shunt Implantation

In our study, the *p*-tau levels increased significantly after shunt surgery. The level of *p*-tau after shunt was 52.1 ± 30.2 pg/mL in the PPV/+SL group and 77.0 ± 41.0 pg/mL in the PPV/+GV group (*P* = .015). This finding is consistent with those of previous reports by Moriya et al^9^ and Tarnaris et al.^34^ Pyykkö et al^35^ measured *p*-tau (Innotest phospho tau-181p; Innogenetics) in the lumbar subarachnoid and intraventricular CSF of patients with iNPH before shunt and found that the mean lumbar and ventricular levels of *p*-tau were 39.5 ± 15.7 pg/mL and 81.3 ± 75.5 pg/mL, respectively. The mean ventricular levels of *p*-tau were twice as high as that in the lumbar subarachnoid space. Collectively, these results support that *p*-tau in the CSF may spread into the subarachnoid space after shunt treatment, although the exact mechanisms have not yet been established. It remains unclear why elevated *p*-tau concentrations occur following CSF drainage. For many years, tau protein was thought to be localized and functional in the cytoplasm, though recent studies have reported that some tau protein may be secreted outside the cell under certain physiological conditions.^36^ Studies have also reported that tau protein can be detected in various cell culture supernatants, such as cultured cells that overexpress tau protein,^37^ primary cultured neurons,^38,39^ and neurons derived from induced pluripotent stem cells.^40^ These forms of extracellular tau protein secretion are different from the nonspecific release of cellular proteins due to cell death and suggest that a specific secretion mechanism may exist. Future research should investigate the presence of tau protein with an aggregation state that is involved in its extracellular release under certain physiological conditions, the participating metabolic machinery, and cell-to-cell transmission/propagation.^41^

### Limitations

This study has some limitations related to its design. First, this was a retrospective cohort study, and is thus inherently prone to selection bias. Second, clinical improvement is a subjective measure that can be difficult to precisely quantify based on patient notes in medical records. Finally, the small sample size limited the power of the study. Future prospective studies are required to verify our findings.

## CONCLUSION

This is the first study to examine LPS with the insertion of a GV in tandem with a PPV for the treatment of iNPH. Our results suggest that CSF shunt flow volume is more restricted in the standing position and is maintained in the supine position, thus improving iNPH symptoms. This may reduce complications associated with intracranial CSF hypotension.

## Notes

**Operative Neurosurgery Speaks!** Audio abstracts available for this article at www.operativeneurosurgery-online.com.

## COMMENT

Utilization of ventriculoperitoneal (VP) shunts for idiopathic normal pressure hydrocephalus (iNPH) has overshadowed lumboperitoneal (LP) shunts, due to historically higher rates of shunt malfunction.^1, 2^ Although randomized clinical trials have comprehensively explored optimal valve settings in VP shunts for iNPH, optimizing LP shunts has lagged far behind.^3^ Thus, the authors should be commended for valiantly chartering into an ill-defined practice in NPH surgery: the optimal LP shunt configuration. This retrospective study of 115 patients compared programmable pressure valves (PPV) with small-lumen (SL) abdominal catheter versus PPV with gravitational “add-on” valve system (GV) in tandem. Following propensity score matching, PPV/+SL fared higher postoperative modified Rankin Scores (mRS) and iNPH grading scale (iNPHGS) gait disturbances compared to the PPV/+GV at 12-month follow up. While the conclusions—“more hardware, ergo better outcomes”—sound more like a talking point from industry representatives, the GV did statistically improve postoperative outcomes. When appropriately matched to historical controls, the add-on valve trended to a lower rate of nonserious adverse events, including subdural hematomas, putatively because the GV suppresses flow volume rate in the standing position to prevent intracranial hypotension. However, some of their improvement in outcome measures may reflect refinement in their techniques over time. Lastly, the GV valve tacked to the fascia in a vertical configuration may have reduced the rate of catheter kinking.

The safety and efficacy of the add-on devices have been validated in the Shunt Valves plus shunt Assistant versus Shunt valves alone for controlling Overdrainage in idiopathic Normal pressure hydrocephalus in Adults (SVASONA) randomized clinical trial.^4^ The GV compared to PPV decreased subdural effusions, overdrainage, and underdrainage at 12-months. However, the study was limited to ventriculoperitoneal shunts, and the allocation groups included either PPV or GV, not PPV in tandem with PPV (PPV/+GV) as in the present review. Nevertheless, long-term outcome studies with gravitational shunts maintain a low complication rate: 3% catheter dislocation/fracture, 5% underdrainage, and 9% overdrainage according to a 5-year follow-up study in VP shunting.^5^ We look forward to similar long-term studies with GV in LP shunts.

**Mohamed Macki**

**Jason Schwalb**

*Detroit, Michigan*

## Operative Neurosurgery Speaks (Audio Abstracts)

Listen to audio translations of this paper's abstract into select languages by choosing from one of the selections below.

## References

[1] Adams RD , Fisher CM, Hakim S, Ojemann RG, Sweet WH. Symptomatic occult hydrocephalus with normal cerebrospinal-fluid pressure. N Engl J Med. 1965;273(3):117–126. 14303656 10.1056/NEJM196507152730301

[2] Hakim S , Adams RD. The special clinical problem of symptomatic hydrocephalus with normal cerebrospinal fluid pressure. J Neurol Sci. 1965;2(4):307–327. 5889177 10.1016/0022-510x(65)90016-x

[3] Pinto FC , Saad F, Oliveira MF Role of endoscopic third ventriculostomy and ventriculoperitoneal shunt in idiopathic normal pressure hydrocephalus. Neurosurgery. 2013;72(5):845–854. 23313977 10.1227/NEU.0b013e318285b37c

[4] Johanson CE , Duncan JA 3rd, Klinge PM, Brinker T, Stopa EG, Silverberg GD. Multiplicity of cerebrospinal fluid functions: new challenges in health and disease. Cerebrospinal Fluid Res. 2008;5(1):10. 18479516 10.1186/1743-8454-5-10 PMC2412840

[5] Iliff JJ , Wang M, Liao Y A paravascular pathway facilitates CSF flow through the brain parenchyma and the clearance of interstitial solutes, including amyloid. Sci Transl Med. 2012;4(147):147ra111–147ra111. 10.1126/scitranslmed.3003748 PMC3551275 22896675

[6] Louveau A , Harris TH, Kipnis J. Revisiting the mechanisms of CNS immune privilege. Trends Immunol. 2015;36(10):569–577. 26431936 10.1016/j.it.2015.08.006 PMC4593064

[7] Lee H , Xie L, Yu M The effect of body posture on brain glymphatic transport. J Neurosci. 2015;35(31):11034–11044. 26245965 10.1523/JNEUROSCI.1625-15.2015 PMC4524974

[8] Xie L , Kang H, Xu Q Sleep drives metabolite clearance from the adult brain. Science. 2013;342(6156):373–377. 24136970 10.1126/science.1241224 PMC3880190

[9] Moriya M , Miyajima M, Nakajima M, Ogino I, Arai H. Impact of cerebrospinal fluid shunting for idiopathic normal pressure hydrocephalus on the amyloid cascade. PLoS One. 2015;10(3):e0119973. 25821958 10.1371/journal.pone.0119973 PMC4379026

[10] Nakajima M , Miyajima M, Ogino I Cerebrospinal fluid biomarkers for prognosis of long-term cognitive treatment outcomes in patients with idiopathic normal pressure hydrocephalus. J Neurol Sci. 2015;357(1-2):88–95. 26169158 10.1016/j.jns.2015.07.001

[11] Bergsneider M , Black PM, Klinge P, Marmarou A, Relkin N. Surgical management of idiopathic normal-pressure hydrocephalus. Neurosurgery. 2005;57(3 suppl):S29–S39. 16160427 10.1227/01.neu.0000168186.45363.4d

[12] Nakajima M , Miyajima M, Ogino I Use of external lumbar cerebrospinal fluid drainage and lumboperitoneal shunts with Strata NSC valves in idiopathic normal pressure hydrocephalus: a single-center experience. World Neurosurg. 2015;83(3):387–393. 25108293 10.1016/j.wneu.2014.08.004

[13] Kazui H , Miyajima M, Mori E Lumboperitoneal shunt surgery for idiopathic normal pressure hydrocephalus (SINPHONI-2): an open-label randomised trial. Lancet Neurol. 2015;14(6):585–594. 25934242 10.1016/S1474-4422(15)00046-0

[14] Miyajima M , Kazui H, Mori E, Ishikawa M, SINPHONI-2 Investigators. One-year outcome in patients with idiopathic normal-pressure hydrocephalus: comparison of lumboperitoneal shunt to ventriculoperitoneal shunt. J Neurosurg. 2016;125(6):1483–1492. 26871203 10.3171/2015.10.JNS151894

[15] Freimann FB , Sprung C. Shunting with gravitational valves–can adjustments end the era of revisions for overdrainage-related events?: clinical article. J Neurosurg. 2012;117(6):1197–1204. 22998061 10.3171/2012.8.JNS1233

[16] Freimann FB , Ötvös J, Chopra SS, Vajkoczy P, Wolf S, Sprung C. Differential pressure in shunt therapy: investigation of position-dependent intraperitoneal pressure in a porcine model. J Neurosurg Pediatr. 2013;12(6):575–581. 24093588 10.3171/2013.8.PEDS13205

[17] Meier U , Stengel D, Müller C Predictors of subsequent overdrainage and clinical outcomes after ventriculoperitoneal shunting for idiopathic normal pressure hydrocephalus. Neurosurgery. 2013;73(6):1054–1060. 24257332 10.1227/NEU.0000000000000155

[18] Miyake H . Shunt devices for the treatment of adult hydrocephalus: recent progress and characteristics. Neurol Med Chir (Tokyo). 2016;56(5):274–283. 27041631 10.2176/nmc.ra.2015-0282 PMC4870182

[19] Miyake H , Ohta T, Kajimoto Y, Nagao K. New concept for the pressure setting of a programmable pressure valve and measurement of in vivo shunt flow performed using microflow meter. J Neurosurg. 2000;92(1):181–187. 10.3171/jns.2000.92.1.0181 10616100

[20] Bergsneider M , Yang I, Hu X, McArthur DL, Cook SW, Boscardin WJ. Relationship between valve opening pressure, body position, and intracranial pressure in normal pressure hydrocephalus: paradigm for selection of programmable valve pressure setting. Neurosurgery. 2004;55(4):851–859. 15458593 10.1227/01.neu.0000137631.42164.b8

[21] Farahmand D , Qvarlander S, Malm J, Wikkelsö C, Eklund A, Tisell M. Intracranial pressure in hydrocephalus: impact of shunt adjustments and body positions. J Neurol Neurosurg Psychiatry. 2015;86(2):222–228. 24963125 10.1136/jnnp-2014-307873

[22] Diesner N , Freimann F, Clajus C, Kallenberg K, Rohde V, Stockhammer F. Female gender predisposes for cerebrospinal fluid overdrainage in ventriculoperitoneal shunting. Acta Neurochir. 2016;158(7):1273–1278. 27177735 10.1007/s00701-016-2827-z

[23] Lemcke J , Meier U, Müller C Safety and efficacy of gravitational shunt valves in patients with idiopathic normal pressure hydrocephalus: a pragmatic, randomised, open label, multicentre trial (SVASONA). J Neurol Neurosurg Psychiatry. 2013;84(8):850–857. 23457222 10.1136/jnnp-2012-303936 PMC3717598

[24] Ishikawa M . Guideline Committee for Idiopathic Normal Pressure Hydrocephalus, Japanese Society of Normal Pressure Hydrocephalus. Clinical guidelines for idiopathic normal pressure hydrocephalus. Neurol Med Chir (Tokyo). 2004;44(4):222–223. 15185767 10.2176/nmc.44.222

[25] Mori E , Ishikawa M, Kato T Guidelines for management of idiopathic normal pressure hydrocephalus: second edition. Neurol Med Chir (Tokyo). 2012;52(11):775–809. 23183074 10.2176/nmc.52.775

[26] van Swieten JC , Koudstaal PJ, Visser MC, Schouten HJ, van Gijn J. Interobserver agreement for the assessment of handicap in stroke patients. Stroke. 1988;19(5):604–607. 3363593 10.1161/01.str.19.5.604

[27] Kubo Y , Kazui H, Yoshida T Validation of grading scale for evaluating symptoms of idiopathic normal-pressure hydrocephalus. Dement Geriatr Cogn Disord. 2008;25(1):37–45. 18025828 10.1159/000111149

[28] Folstein MF , Folstein SE, McHugh PR. “Mini-mental state”. A practical method for grading the cognitive state of patients for the clinician. J Psychiatr Res. 1975;12(3):189–198. 1202204 10.1016/0022-3956(75)90026-6

[29] Dubois B , Slachevsky A, Litvan I, Pillon B. The FAB: a Frontal Assessment Battery at bedside. Neurology. 2000;55(11):1621–1626. 11113214 10.1212/wnl.55.11.1621

[30] Miyajima M , Nakajima M, Ogino I, Miyata H, Motoi Y, Arai H. Soluble amyloid precursor protein alpha in the cerebrospinal fluid as a diagnostic and prognostic biomarker for idiopathic normal pressure hydrocephalus. Eur J Neurol. 2013;20(2):236–242. 22672777 10.1111/j.1468-1331.2012.03781.x

[31] Kajimoto Y , Ohta T, Miyake H Posture-related changes in the pressure environment of the ventriculoperitoneal shunt system. J Neurosurg. 2000;93(4):614–617. 11014539 10.3171/jns.2000.93.4.0614

[32] Kaijima M , Fukuda H, Yamamoto K. Post-operative complications peculiar to lumboperitoneal shunt: possible consequences due to side leakage of CSF from around the inserted spinal tube into the lumbar epidural space [in Japanese]. No Shinkei Geka. 2011;39(5):497–504. 21512201

[33] Matsubara T , Ishikawa E, Hirata K A new mechanism of cerebrospinal fluid leakage after lumboperitoneal shunt: a theory of shunt side hole–case report. Neurol Med Chir (Tokyo). 2014;54(7):572–577. 24305015 10.2176/nmc.cr.2013-0067 PMC4533463

[34] Tarnaris A , Toma AK, Chapman MD, Keir G, Kitchen ND, Watkins LD. Use of cerebrospinal fluid amyloid-beta and total tau protein to predict favorable surgical outcomes in patients with idiopathic normal pressure hydrocephalus. J Neurosurg. 2011;115(1):145–150. 21438653 10.3171/2011.2.JNS101316

[35] Pyykkö OT , Lumela M, Rummukainen J Cerebrospinal fluid biomarker and brain biopsy findings in idiopathic normal pressure hydrocephalus. PLoS One. 2014;9(3):e91974. 24638077 10.1371/journal.pone.0091974 PMC3956805

[36] Simón D , García-García E, Gómez-Ramos A Tau overexpression results in its secretion via membrane vesicles. Neurodegener Dis. 2012;10(1-4):73–75. 22269430 10.1159/000334915

[37] Chai X , Dage JL, Citron M. Constitutive secretion of tau protein by an unconventional mechanism. Neurobiol Dis. 2012;48(3):356–366. 22668776 10.1016/j.nbd.2012.05.021

[38] Kanmert D , Cantlon A, Muratore CR C-terminally truncated forms of tau, but not full-length tau or its C-terminal fragments, are released from neurons independently of cell death. J Neurosci. 2015;35(30):10851–10865. 26224867 10.1523/JNEUROSCI.0387-15.2015 PMC6605107

[39] Karch CM , Jeng AT, Goate AM. Extracellular Tau levels are influenced by variability in Tau that is associated with tauopathies. J Biol Chem. 2012;287(51):42751–42762. 23105105 10.1074/jbc.M112.380642 PMC3522274

[40] Bright J , Hussain S, Dang V Human secreted tau increases amyloid-beta production. Neurobiol Aging. 2015;36(2):693–709. 25442111 10.1016/j.neurobiolaging.2014.09.007

[41] Frost B , Jacks RL, Diamond MI. Propagation of tau misfolding from the outside to the inside of a cell. J Biol Chem. 2009;284(19):12845–12852. 19282288 10.1074/jbc.M808759200 PMC2676015

